# Patterns of Inpatient Admissions during *Hajj*: Clinical conditions, length of stay and patient outcomes at an advanced care centre in Makkah, Saudi Arabia

**DOI:** 10.12669/pjms.344.15989

**Published:** 2018

**Authors:** Ahmad A. Mirza, Mohammed A. Al-Sakkaf, Amrallah A. Mohammed, Mian U. Farooq, Ziad A. Al-Ahmadi, Mohammed A. Basyuni

**Affiliations:** 1 Ahmad A. Mirza Department of Otolaryngology, Head and Neck Surgery, Faculty of Medicine in Rabigh, King Abdulaziz University, Jeddah, Saudi Arabia; 2 Mohammed A. Al-Sakkaf, Department of Surgery, Security Forces Hospital Program, Makkah, Saudi Arabia; 3 Amrallah A. Mohammed Departments of Home Health Care, King Abdullah Medical City, akkah, Saudi Arabia. Department of Medical Oncology, Faculty of Medicine, Zagazig University, Zagazig, Egypt; 4 Mian U. Farooq Department of Strategic Planning and Institutional Advancement, King Abdullah Medical City, Makkah, Saudi Arabia; 5 Ziad A. Al-Ahmadi, Faculty of Medicine, Umm Al-Qura University, Makkah, Saudi Arabia; 6 Mohammed A. Basyuni, Faculty of Medicine, Umm Al-Qura University, Makkah, Saudi Arabia

**Keywords:** Inpatients, Patient Admission, Length of Stay, Quality Improvement, Tertiary Healthcare, Saudi Arabia

## Abstract

**Objectives:**

This study aimed to describe inpatient clinical conditions at an advanced care facility in Saudi Arabia during the annual *Hajj* pilgrimage and to determine factors correlating with length of stay (LOS).

**Methods:**

This retrospective study was conducted at the King Abdullah Medical City (KAMC), Makkah, Saudi Arabia, and included all inpatients admitted during the annual *Hajj* pilgrimage between August and October 2015. Demographic, administrative and clinical data were collected from patient charts and analysed.

**Results:**

A total of 296 inpatients were included in the study, of which the majority were male (73.6%) and ≥55 years old (77%). Walk-in admissions occurred less frequently than referrals (38.9% versus 61.1%). Most patients (41.6%) were admitted during the peak *Hajj* period (the 8-13^th^ days of *Dhu al-Hijjah*). Acute coronary syndrome was the most prevalent provisional diagnosis (65.2%). In terms of outcomes, 89.2% of the inpatients were discharged in a stable condition, with 37.5% discharged within ≤24 hours of admission. However, 39.9% required admission to the Intensive Care Unit (ICU). Overall, LOS was significantly associated with various factors, including the mode of admission, admission period, admitting department, number of comorbidities and ICU admission (*P* <0.050 each).

**Conclusion:**

Most of admissions were referrals, and the main *Hajj* period witnessed the majority of admissions. The vast majority of inpatients eventually discharged in a stable condition. Determinants of the length of hospital stay were the mode of admission, admission period, admitting department, number of comorbidities and ICU admission.

## INTRODUCTION

The annual *Hajj* pilgrimage is a compulsory requirement for members of the Islamic faith who are physically and financially able to make the journey; it involves travelling to Makkah, the capital of Saudi Arabia, during the month of *Dhu al-Hijjah*, which is the last month of the *Hijri* calendar.^1^,^2^ Some pilgrims arrive in Makkah nearly one month before the central rituals of *Hajj*, which begin officially on the 8–13^th^ day of *Dhu al-Hijjah*, and often stay for another month after the pilgrimage ends. The annual *Hajj* pilgrimage is considered one of the largest annual mass gatherings in the world.^3^ Furthermore, the pilgrimage itself involves taking part in an array of physically demanding rituals at specific locations and during short periods of time.^4^

The risk of heat exhaustion as well as the spread of communicable diseases is greatly increased during Hajj.^4^–^6^ Moreover, people with chronic diseases and the elderly are at greater risk of medical complications.^7^-^10^ Common diagnoses among hospitalised pilgrims include cardiac conditions and respiratory infections such as pneumonia.^7^,^10^,^11^ In addition, inpatients also frequently suffer from comorbidities, including hypertension, diabetes mellitus, cardiac conditions and chronic lung diseases.^10^-^13^

In Makkah, the healthcare system is composed of secondary and tertiary medical centres which provide high-quality care to patients during the *Hajj* period. A recent study conducted at two medical centres near the Al-Haram Holy Mosque found that 16.5% of pilgrims required further medical treatment in an advanced tertiary healthcare centre.^14^ Similarly, another study evaluating hospitalisation and patient admissions in Makkah concluded that about 3% of patients required admission to a tertiary hospital.^15^ The Ministry of Health in Saudi Arabia recently established the King Abdullah Medical City (KAMC) in Makkah as a response to increased demands on tertiary healthcare services in the region. Nevertheless, few studies have evaluated patterns of admission or length of stay (LOS) in secondary care hospitals and none has been conducted at the ultimate healthcare facility in the region, KAMC.^10^-^13^ Thus, the current study aimed to describe admission patterns and inpatient outcomes at the KAMC during the annual *Hajj* pilgrimage.

## METHODS

This retrospective cross-sectional study was conducted at the KAMC and included all inpatients admitted between the 16^th^ day of *Dhu al-Qidah* and the fourth day of *Muharram*, corresponding to 31 August–17 October 2015. This particular *Hajj* season was divided into three consecutive periods—the pre-ritual period before the 8^th^ day of *Dhu al-Hijjah* (21 days), the peak *Hajj* period between the 8–13^th^ days of *Dhu al-Hijjah* (six days) and the post-ritual period after the 13^th^ day of *Dhu al-Hijjah* (21 days). The inpatients’ demographic (i.e. age, gender and nationality), admission (i.e. the admitting department, admission mode and date and time of admission) and clinical (i.e. provisional diagnostic category, evidence of infection, admission to the Intensive Care Unit [ICU] and the presence of chronic diseases, complications or comorbidities) data were collected from hospital charts using Medica Plus software.

During the study period, admission to the ICU occurred as a result of significant in hospital complications such as cases requiring mechanical ventilation or those suffering from sepsis, shock, acute kidney injuries or severe electrolyte imbalance. Comorbidities were defined as the presence of diabetes mellitus, hypertension, Ischaemic Heart Disease (IHD), hyperlipidaemia or chronic obstructive pulmonary diseases. The overall LOS was calculated from the time of admission until discharge. Inpatients were categorised according to mode of admission as either walk-ins (i.e. those presenting to the emergency room) or referrals (i.e. those transferred to the KAMC from other healthcare centres). Provisional diagnoses were divided into five categories: acute coronary syndrome (ACS), altered levels of consciousness, pulmonary oedema, a heart blockage or other. Patient outcomes were divided into four categories as either improved, deceased, absconded/discharged against medical advice or transferred to their home country.

Data were analysed using the Statistical Package for the Social Sciences (SPSS), Version 16.0 (IBM Corp., Armonk, New York, USA). Binary variables included gender (male or female), mode of admission (walk-in or referral), bacterial blood culture results (positive or negative) and ICU admission (yes or no). Multinomial variables included the inpatient’s nationality, number of comorbidities, admitting department, admission period, provisional diagnostic category and outcome. Binary variables were compared using the Mann-Whitney U-test, while a Kruskal-Wallis H-test was used to compare multinomial variables. Age and LOS were treated as categorical variables. A Shapiro-Wilk analysis was conducted to determine if the distribution of the data was normal. Non-parametric data were expressed as medians and Interquartile Ranges (IQRs) at the 25^th^ and 75^th^ percentiles. Spearman’s correlation coefficient was used to calculate the null hypothesis which stated that there would be no relationship between age and LOS. An alpha level of *P* <0.050 was considered significant.

Ethical approval to conduct the study was provided by the Institutional Review Board (IRB) of the KAMC prior to data collection.

## RESULTS

A total of 306 inpatients were admitted to the KAMC during the study period; however, 10 patients (3.3%) were excluded due to incomplete data. Therefore, a total of 296 inpatients were included in the study. None were of Saudi nationality and the majority were male (73.6%). The male-to-female ratio was approximately 3:1. Patients aged 55–64 years old were most frequently admitted (39.2%), followed by those aged 65–74 years old (26.7%). The median age was 61 years (IQR: 55–69 years old). Most cases were referred from other healthcare facilities (61.1%) and were admitted during the peak *Hajj* period (41.6%). The most frequent provisional diagnosis was ACS (65.2%), followed by an altered level of consciousness (14.2%). Correspondingly, the majority of patients were admitted to the cardiology department (78.4%).

A total of 72 patients (24.3%) had a single comorbidity, while 67 (22.6%) and 108 (36.5%) patients had two, three or more comorbidities, respectively. There were 118 inpatients (39.9%) admitted to the ICU. According to their bacterial blood culture results, 9.5% of the patients developed an in-hospital infection. The LOS for most inpatients was ≤ 5 days (72.3%), with 37.5% staying ≤ 1 day. In terms of patient outcomes, there were very few deaths (3.7%). The vast majority showed clinical improvement and were subsequently discharged in a stable condition (89.2%) [Table-I].

**Table-I:** Demographic, admission and clinical characteristics of inpatients admitted to the King Abdullah Medical City, Makkah, Saudi Arabia, during an annual Hajj pilgrimage (N = 296).

Characteristic	n (%)
**Age in years**	
23–34	4 (1.4)
35–44	15 (5.1)
45–54	49 (16.6)
55–64	116 (39.2)
65–74	79 (26.7)
75–84	30 (10.1)
≥85	3 (1)
**Gender**	
Male	218 (73.6)
Female	78 (26.4)
**Nationality**	
Indian	48 (16.2)
Bangladeshi	30 (10.1)
Pakistani	29 (9.8)
Egyptian	26 (8.8)
Iranian	15 (5.1)
Indonesian	16 (5.4)
Other Arab country*	132 (44.6)
**Mode of admission**	
Walk-in	115 (38.9)
Referral	181 (61.1)
**Admission period**	
Pre-ritual	115 (38.9)
Hajj	123 (41.6)
Post-ritual	58 (19.6)
**Admitting department**	
Cardiology	232 (78.4)
Cardiothoracic surgery	28 (9.5)
General internal medicine	23 (7.8)
Other^†^	13 (4.4)
**Provisional diagnostic category**	
ACS^‡^	193 (65.2)
Altered level of consciousness	42 (14.2)
Pulmonary oedema	16 (5.4)
Heart blockage	15 (5.1)
Other	30 (10.1)
**Number of comorbidities^§^**	
None	49 (16.6)
1	72 (24.3)
2	67 (22.6)
≥3	108 (36.5)
**Blood culture results**	
Positive	28 (9.5)
Negative	268 (90.5)
**ICU admission**	
Yes	118 (39.9)
No	178 (60.1)
LOS in days	
≤1	111 (37.5)
2–5	103 (34.8)
6–9	27 (9.1)
10–13	16 (5.4)
≥14	39 (13.2)
**Patient outcome**	
Died	11 (3.7)
Improved	264 (89.2)
Absconded/DAMA	18 (6.1)
Transferred to home country	3 (1)

ACS = acute coronary syndrome; ICU = intensive care unit; LOS = length of stay; DAMA = discharged against medical advice.

* Not including Saudi Arabia.

† Including haematology, ear, nose and throat, ophthalmology, neuroscience and other surgical specialties.

‡ Including ST-segment elevation and non-ST-segment elevation myocardial infarctions and unstable angina.

§ Including diabetes, hypertension or pulmonary diseases.

The LOS was not found different among different age groups and gender, i.e. p = 0.809 and 0.343, respectively. However, the median LOS for referred cases was significantly higher than that of walk-in cases (median: 3 days, IQR: 1–7 days versus median: 2 days, IQR: 1–6 days; *P* = 0.014). A significant difference in LOS was also observed between patients admitted to different specialties (*P* <0.001) and those admitted during different admission periods (*P* = 0.023) [Table-II].

**Table-II:** Factors influencing length of stay among inpatients admitted to the King Abdullah Medical City, Makkah, Saudi Arabia, during an annual Hajj pilgrimage (N = 296).

Characteristic	Median LOS in days (IQR)	Mean rank	P value*
**Gender**			0.343
Male	2 (1–7)	145.8	
Female	3 (1–7)	156.2	
**Nationality**			0.604
Indian	3 (1–10)	157.5	
Bangladeshi	3 (2–6)	160.7	
Pakistani	2 (1–10)	148	
Egyptian	2 (1–5)	133.8	
Iranian	2 (1–4)	116.4	
Indonesian	3 (1–9)	154.7	
Other Arab country†	2 (1–7)	147.1	
**Mode of admission**			0.014
Walk-in	2 (1–6)	133.6	
Referral	3 (1–7)	158	
**Admission period**			0.023
Pre-ritual	2 (1–6)	138.9	
Hajj	2 (1–6)	143.3	
Post-ritual	3 (2–8)	171.4	
**Admitting department**			<0.001
Cardiology	2 (1–4)	129.6	
Cardiothoracic surgery	12 (10–19)	252.3	
General internal medicine	6 (3–11)	201.1	
Other‡	3 (2–7)	176.3	
**Provisional diagnostic category**			<0.001
ACS§	1 (1–3)	118	
Altered level of consciousness	13 (4–26)	224.9	
Pulmonary oedema	4 (3–10)	196.2	
Heart blockage	3 (1–4)	137.2	
Other	5 (3–9)	185.2	
Number of comorbidities¶			<0.001
None	1 (1–3)	104	
1	1 (1–3)	108	
2	2 (1–6)	154.7	
≥3	5 (2–14)	191	
**Blood culture results**			<0.001
Positive	15 (8–25)	248.7	
Negative	2 (1–5)	135.7	
**ICU admission**			<0.001
Yes	9 (4–19)	222.8	
No	1 (1–2)	92.2	
**Patient outcome**			0.095
Died	6 (2–33)	200.7	
Improved	2 (1–6.5)	147.1	
Absconded/DAMA	2 (1–6.5)	138.1	

LOS = length of stay; IQR = interquartile range; ACS = acute coronary syndrome;

ICU = intensive care unit; DAMA = discharged against medical advice.

* Using the Mann-Whitney U-test for binomial variables and Kruskal-Wallis H-test for multinomial variables.

† Not including Saudi Arabia.

‡ Including haematology, ear, nose and throat, ophthalmology, neuroscience and other surgical specialties.

§ Including ST-segment elevation and non-ST-segment elevation myocardial infarctions and unstable angina.

¶ Including diabetes, hypertension or pulmonary diseases.

## DISCUSSION

Currently, there are two main modes of admission at the KAMC during the *Hajj* period. Referred patients receive customised treatment by highly-qualified physicians while 24-hour emergency services are available on a walk-in basis. In the current study, referrals from other healthcare facilities were found to constitute the majority of hospital admissions; however, walk-in admissions were also fairly common. This is in accordance with previous research conducted at the Al-Noor Specialist Hospital, another advanced healthcare centre in the region.^10^ Most secondary healthcare centres located in Makkah and Al-Mashaer are well-equipped for emergency situations and to provide short-term hospitalisation services. Furthermore, patients admitted to such centres may be transferred to other secondary or tertiary hospitals for longer-term hospitalisation within the last few days of *Hajj*. Subsequently, patients in stable condition are followed up at secondary care hospitals until they are discharged, whereupon they are seen at the primary healthcare centre located in their respective residential camps.^10^,^12^,^13^

As per previous research, the findings of the present study indicated that the primary *Hajj* period (the 8–13^th^ days of *Dhu al-Hijjah*) was the time during which most patients sought healthcare.^10^ Moreover, the vast majority of patients admitted to the KAMC were ≥45 years of age, with 82.4% aged between 45–75 years old. An earlier study similarly found that 68.6% of admitted patients at the Al-Noor Specialist Hospital were 60 years of age or older.^10^ In another study conducted in temporary health facilities in Mina and Arafat, most admitted cases were over 40 years old (79%).^12^ As the financial demands of the *Hajj* pilgrimage are relatively costly, most *Hajj* visitors are usually elderly because such individuals are more likely to be able to afford such costs. However, another study conducted in two small healthcare centres near the Al-Haram Holy Mosque reported that the ages of *Hajj* pilgrims were more evenly distributed.^14^

As most *Hajj* visitors are elderly, they are more vulnerable to developing serious medical complications such as IHD; this may explain why the highest proportion of patients in the current study were admitted to the cardiac department and provisionally diagnosed with ACS. In contrast, the findings of two other studies of secondary hospitals in Al-Mashaer and Mina/Arafat revealed that respiratory tract infections such as pneumonia were the leading cause of admission (39.4% and 19.7%, respectively), followed by cardiovascular events like IHD (8.8% and 12.3%, respectively).^13^,^14^ However, the relatively cooler weather during the 2002 and 2003 *Hajj* pilgrimages—which fell in those years during the month of February—may have played a role as an effect modifier in these cases. In contrast, Hasan *et al*. found that ACS was the primary cause of admissions among known cases of diabetes during the *Hajj* period.^16^

In terms of clinical outcomes, approximately 40% of inpatients in the current study were transferred to the ICU. These findings were extremely high compared to those reported by Khan *et al*. (25.5%) and Madani *et al*. (9.4%).^10^,^12^ This variation might be due to the higher proportion of patients in these studies who had been admitted to medical departments, thereby excluding surgical patients.^10^,^12^ Nevertheless, despite the higher rate of admission to the ICU in the current study, there were very few fatalities (3.7%), with almost 90% of patients showing improvement and being discharged in good condition. This suggests that the KAMC provides a high standard of medical care during the *Hajj* pilgrimage. In contrast, Khan *et al*. reported a higher incidence of mortality during their study (16.5%).^10^ In a previous study of the admission patterns of seven hospitals in Al-Mashaer, including KAMC, Al-Ghamdi *et al*. reported that 40% of admitted patients were discharged in stable condition, while 59% were transferred to other secondary or tertiary hospitals.^13^ Such findings are an indicator that the healthcare facilities in this region are highly standardised and provide both short- and long-term hospitalisation. However, in view of the high proportion of patients who were discharged within the first 24 hours in the current study, further longitudinal studies are recommended to investigate whether some patients are admitted unnecessarily.

In the present study, LOS was found to be strongly associated with various factors, including referral from another healthcare facility, admission during the post-ritual period, admission to the cardiothoracic surgery department, being provisionally diagnosed with an altered level of consciousness, having three or more comorbidities, evidence of bacterial infection and admission to the ICU. On the other hand, age and nationality showed no relationship with LOS.

There are several potential limitations to the current study. First, the data collected was incomplete due to a lack of patient-reported information in the hospital records. Second, the analysis included individuals of diverse backgrounds and characteristics; nevertheless, the findings of the study could be relevant to other healthcare systems that cater to mass gatherings with large numbers of individuals of different nationalities. Lastly, due to the lunar nature of the *Hijri* calendar, the Islamic year is nearly 11 days shorter than that of the Gregorian calendar which thus signifies that weather conditions during the annual *Hajj* pilgrimage may vary. This phenomenon partially limits the generalisation of the current findings to other *Hajj* pilgrimages as well as other rituals based on the *Hijri* calendar.

## CONCLUSION

Referral cases were the preponderance and the majority of admissions occurred during the main hajj period. While most of admitted cases discharged eventually in a stable status, just more than a third of them discharged within the first 24 hours of admission. Admissions of Referral, during post-hajj period, to ICU and cardiothoracic surgery service, with more than two comorbidities, with positive blood culture and altered level of consciousness are associated with relatively longer stay in the hospital. These data may be helpful to hospital administrators and policy-makers in providing cost-effective healthcare to pilgrims.

## References

[1] Gatrad AR, Sheikh A (2005). Hajj Journey of a lifetime. BMJ.

[2] Al-Jasser FS, Kabbash IA, Almazroa MA, Memish ZA (2012). Patterns of diseases and preventive measures among domestic hajjis from Central, Saudi Arabia. Saudi Med J.

[3] Shujaa A, Alhamid S (2015). Health response to Hajj mass gathering from emergency perspective, narrative review. Turk J of Emerg Med.

[4] Shafi S, Memish ZA, Gatrad AR, Sheikh A (2005). Hajj 2006:Communicable disease and other health risks and current official guidance for pilgrims. Euro Surveill.

[5] Ahmed QA, Arabi YM, Memish ZA (2006). Health risks at the Hajj. Lancet.

[6] Khogali M (1983). Epidemiology of heat illnesses during the Makkah pilgrimages in Saudi Arabia. Int J Epidemiol.

[7] Alzeer A, Mashlah A, Fakim N, Al-Sugair N, Al-Hedaithy M, Al-Majed S (1998). Tuberculosis is the commonest cause of pneumonia requiring hospitalization during Hajj (pilgrimage to Makkah). J Infect.

[8] Novelli VM, Lewis RG, Dawood ST (1987). Epidemic group A meningococcal disease in Haj pilgrims. Lancet.

[9] Balkhy HH, Memish ZA, Bafaqeer S, Almuneef MA (2004). Influenza a common viral infection among Hajj pilgrims:Time for routine surveillance and vaccination. J Travel Med.

[10] Khan NA, Ishag AM, Ahmad MS, El-Sayed FM, Bachal ZA, Abbas TG (2006). Pattern of medical diseases and determinants of prognosis of hospitalization during 2005 Muslim pilgrimage Hajj in a tertiary care hospital:A prospective cohort study. Saudi Med J.

[11] Yousuf M, Al-Saudi DA, Sheikh RA, Lone MS (1995). Pattern of medical problems among Haj pilgrims admitted to King Abdul Aziz Hospital, Madinah Al-Munawarah. Ann Saudi Med.

[12] Madani TA, Ghabrah TM, Al-Hedaitzhy MA, Alhazmi MA, Alazraqi TA, Albarrak AM (2006). Causes of hospitalization of pilgrims in the Hajj season of the Islamic year 1423 (2003). Ann Saudi Med.

[13] Al-Ghamdi SM, Akbar HO, Qari YA, Fathaldin OA, Al-Rashed RS (2003). Pattern of admission to hospitals during Muslim pilgrimage (Hajj). Saudi Med J.

[14] Bakhsh AR, Sindy AI, Baljoon MJ, Dhafar KO, Gazzaz ZJ, Baig M (2015). Diseases pattern among patients attending Holy Mosque (Haram) medical centers during Hajj 1434 (2013). Saudi Med J.

[15] Sindy AI, Baljoon MJ, Zubairi NA, Dhafar KO, Gazzaz ZJ, Deiab BA (2015). Pattern of patients and diseases during mass transit:The day of Arafat experience. Pak J Med Sci.

[16] Hasan G, Moabber H, Alyamani A, Sayeed A, Altatar F (2016). Study on risk factors (predisposing factors) for poor diabetes control during Hajj (1436/2015) in people with diabetes. Pak J Med Sci.

